# Is there a role for artificial intelligence in end-of-life care for persons with dementia? Perspectives of and recommendations by caregivers and hospice staff

**DOI:** 10.1093/geroni/igag105

**Published:** 2026-09-18

**Authors:** Oonjee Oh, Debra Parker Oliver, Karla Washington, George Demiris

**Affiliations:** School of Nursing, University of Pennsylvania, Philadelphia, Pennsylvania, United States; Perelman School of Medicine, University of Pennsylvania, Philadelphia, Pennsylvania, United States; School of Medicine, Washington University in St. Louis, St. Louis, Missouri, United States; School of Medicine, Washington University in St. Louis, St. Louis, Missouri, United States; School of Nursing, University of Pennsylvania, Philadelphia, Pennsylvania, United States; Perelman School of Medicine, University of Pennsylvania, Philadelphia, Pennsylvania, United States

**Keywords:** Technology, Palliative care, Cognitive impairment

## Abstract

**Background and Objectives:**

Tools powered by artificial intelligence (AI) are reshaping many aspects of dementia care, and end-of-life caregiving for persons with dementia may not be exempt from such paradigm shifts. In this study, we explored families’ and staff’s acceptance of AI in hospice dementia care and generated actionable recommendations for AI designers in supporting end-of-life care for persons with dementia.

**Design and Methods:**

In this qualitative descriptive study, we conducted individual and focus group interviews with family caregivers (*N *= 30) of hospice patients with dementia and hospice staff (*N *= 9). We utilized the Technology Acceptance Model as our theoretical framework and conducted directed content analysis.

**Results:**

Participants described AI tools as potentially helpful for obtaining information, monitoring patients, managing medications and appointments, providing companionship, and conducting logistical tasks. Familiarity with technology, along with user-friendly interfaces and guidance, emerged as subcategories of perceived ease of use. The uniqueness of hospice, encompassing its humanistic nature and emphasis on families as part of the care unit, also emerged as a key factor impacting attitudes towards AI use. Although many anticipated that AI would bring inevitable changes in the future, they showed nuanced trust and perspectives on how it would impact the essence of care. Based on our participants’ narratives, recommendations emerged across three domains: population-related, AI feature-related, and deployment-related.

**Discussion and Implications:**

Families and frontline staff should not be considered merely as end users but as key stakeholders whose needs and expertise should shape the direction of AI applications.

Innovation and Translational Significance: Our study is the first to explore AI integration in hospice dementia care from the perspectives of those at the frontline of care. Participants acknowledged AI’s inevitability and saw potential in it for informational and logistical support, patient monitoring, medication management and care coordination, and companionship, with trust and ease of use essential for adoption. Our actionable recommendations, grounded in stakeholders’ voices, bridge the gap between technological innovation and compassionate end-of-life care. It contributes to offering practical guidance on designing AI integration in vulnerable care settings and strengthening stakeholder involvement to improve acceptability of AI tools.

## Introduction

With the global trend of population growth and aging, the number of persons with dementia is estimated to increase to 153 million cases worldwide by 2050.^1^ While dementia is often characterized as a chronic condition, it is ultimately life-limiting, with patients dying from complications such as pneumonia or feeding difficulties.^2^ In the United States, one out of three older adults die with or from dementia,^3^ making it one of the leading causes of death.^4^ For individuals with a life expectancy of 6 months or less, hospice provides the gold standard of care, offering medical, emotional, and spiritual support to promote comfort.^5^ The National Hospice and Palliative Care Organization reported that 25% of Medicare decedents received hospice primarily due to neurological and dementia-related diagnoses in 2022,^6^ and most hospice agencies provide care for persons with dementia.^7^

Despite the increasing prevalence and impact of dementia in hospice, challenges regarding hospice dementia care persist. For instance, Bosco et al. reported that caregivers of hospice-enrolled persons with dementia experience substantial physical and emotional strain as they engage in intense day-to-day care and end-of-life planning.^8^ Harrison et al. also found that hospice staff often struggle with families not being fully aware of the terminal nature of dementia in their role as proxy decision-makers.^9^ Despite these challenges, hospice interventions specifically tailored to dementia remain limited. Lassell et al. identified only 10 intervention studies targeting hospice dementia care. These interventions included training interventions aimed at improving hospice clinicians’ knowledge and quality of care, add-on services aimed at supporting persons with dementia and caregivers in hospice, and others focusing on nonpharmacological symptom management or goals-of-care decision-making, all of which were in the early stages of development and pilot testing.^10^ This limited evidence highlights the need to identify resources that can better support end-of-life care for persons with dementia, their caregivers, and hospice staff.

Recently, artificial intelligence (AI) has shown great promise in dementia care. With its strength in personalization and automation, many AI tools have been developed to be applied throughout diagnosis, prognosis, and disease management.^11^ Habbal et al. provided an overview of how AI technologies are being used across dementia care, including AI-based smart environments and multimodal tests that can monitor patients and contribute to timely diagnosis of cognitive or functional decline, AI chatbots that can detect symptoms of dementia through conversations, machine learning and big data analytics that optimize therapeutic pipelines in clinical trials and manage patient heterogeneity, and AI models that personalize prescription models.^12^ Although AI has transformed many areas of dementia care, its influence on the end-of-life phase, a critical segment of the care continuum, has been limited. Given hospice’s emphasis on quality of life and person-centered care, integrating AI raises important ethical and practical considerations. Yet, as AI applications continue to expand across healthcare, similar shifts in end-of-life care may be imminent. For instance, algorithms predicting mortality could identify potential hospice candidates, AI-driven chatbots could address the informational and communication needs of hospice caregivers, and clinical decision support systems could inform decision-making and support triage for hospice clinicians.^13^

With the introduction of AI in end-of-life care, patients and care providers, including hospice staff and family caregivers, are the ones directly affected by its implementation. While many professional organizations and experts have made recommendations regarding AI in healthcare and science,^14-^^16^ the voices of those at the frontline of care remain largely unknown, particularly in the end-of-life context. Exploring how caregivers and staff providing care for terminally ill patients with dementia perceive and accept AI can provide crucial insights to guide future directions, ensuring that AI tools are designed to meet the needs of their intended target populations.

Therefore, we aimed to explore caregivers’ and hospice staff’s perspectives on the use of AI across various aspects of hospice dementia care that may include symptom monitoring, decision-making, and caregiver education, and to develop recommendations informed by their narratives. To explore to what extent AI may or may not be accepted in hospice dementia care, our first research question was: 1) What are the attitudes toward AI, perceived usefulness, and perceived ease of use described by caregivers of persons with dementia and hospice staff? The exploration of this research question was guided by the Technology Acceptance Model (TAM).^17^ The TAM posits that users’ behavioral intention, commonly referred to as acceptance within this framework,^18^ is impacted by their attitude toward the technology, which is shaped by perceived usefulness and ease of use.^17^ Based on these insights, our second research question was: 2) What do these stakeholders at the forefront of care perceive as important future directions for AI in hospice dementia care? We sought to offer actionable recommendations for AI integration in end-of-life care settings for persons with dementia grounded in stakeholders’ voices.

## Methods

### Study design

To provide a straightforward description and data-driven interpretation on how caregivers and hospice clinicians perceive the role of AI in hospice dementia care,^19^^,^^20^ we used a qualitative descriptive approach situated within the naturalistic paradigm.^21^^,^^22^ We conducted semi-structured interviews, employing both individual and focus group interviews, with caregivers of persons with dementia and interdisciplinary staff in hospice. Given the scarcity of qualitative work on AI acceptance in end-of-life care for persons with dementia, we conducted directed content analysis and used the TAM as our theoretical framework that guided our interview questions and initial coding scheme.^21^

### Setting and procedures

We recruited caregivers of hospice patients with dementia from three large hospice agencies in the Northeast and Midwest United States as part of a larger randomized controlled trial of an intervention called ENCODE (Empowering Caregivers of Patients with Dementia; ClinicalTrials.gov identifier: NCT05336344).^23^ The eligibility criteria for the ENCODE trial were: 1) enrolled as an informal caregiver of a hospice patient with a dementia-related diagnosis, 2) aged 18 years or older, 3) having concerns about pain management, and 4) able to speak and read English, without cognitive impairment as assessed by the research staff. Among the ENCODE trial participants, we interviewed only those assigned to the attention control (AC) group. Hospice staff were recruited from a Medicare- and Medicaid-certified hospice agency in the Northeast United States. Eligibility criteria were: 1) working in hospice for at least one year and 2) having experience interacting with a hospice patient with dementia.

For caregivers, master's-prepared mental health counselors collected their demographics (e.g., age, sex, and education level) and conducted individual interviews. For hospice staff, the first author, a master's-prepared nurse and PhD candidate, conducted mini focus group interviews of two to four staff members. All protocols were approved by the Institutional Review Board (IRB) at the University of Pennsylvania (#849217, 07/19/2021; #857746, 01/17/2025).

### Data collection

Given differences in roles, care context, and interview format between staff and caregivers, specific interview questions varied slightly (Supplementary Table 1). Nonetheless, both interviews followed similar structures. We began by introducing the definition of AI and hypothetical examples of AI being applied in hospice (e.g., using AI algorithms to screen hospice candidates; AI chatbots providing informational or conversational support to families in hospice; AI-powered technology involved in symptom monitoring, scheduling, or administrative tasks; robots assisting daily activities). This was followed by open-ended questions based on the TAM components.^17^ Beyond these broad examples, participants were invited to identify specific scenarios where AI could support their caregiving or professional tasks. Prior to the start of data collection, the interview guide for caregivers was reviewed by experts in hospice, dementia, bioethics, gerontology, and family caregiving and was also pilot-tested with a randomly selected AC group member; questions were revised as needed for clarity.

For caregivers, the latter half of a one-hour AC group session was dedicated to the study’s interview questions; focus group interviews with hospice staff lasted one hour. All interviews were audio-recorded and transcribed verbatim through a professional transcription service.

### Data analysis

Transcripts from caregiver interviews were analyzed in a random order. Once no new codes or ideas were emerging from the data, five additional transcripts were coded to confirm data saturation. This approach yielded a sample size of 30 caregivers. For the hospice staff, all eligible members who expressed interest were invited to participate; three rounds of focus groups were conducted (*n* = 9). Transcripts were uploaded to Atlas.ti software for data storage and analysis.^24^

Predetermined categories were derived from the TAM (i.e., perceived usefulness, perceived ease of use, attitudes toward using AI, and actual use of AI).^17^ To ensure that the use of TAM would not inadvertently omit aspects related to our phenomenon of interest, we simultaneously examined the data inductively using *in vivo* codes.^21^ Two authors (OO and GD) independently coded six transcripts and met regularly to discuss coding decisions and emerging findings. The remaining transcripts were coded by the first author (OO). We then examined the hierarchical structure of the codes within each deductive and inductive category and inductively identified subcategories that shared meaning. To generate recommendations, we used *in vivo* codes to code excerpts reflecting participants’ thoughts on necessary preparations and ideal approaches for navigating AI, which were subsequently grouped into an inductive category labeled ‘preparing for the future.’ Subcategories identified from this category were translated into actionable recommendations. We maintained audit trails documenting analytic revisions and employed peer debriefing, in which the authorship team reviewed the written work for critical feedback.^25^ Reflexivity statements, including our roles throughout the process and positionality, are presented in Supplementary Table 2.

## Results

This study included 30 caregivers of hospice patients with dementia and 9 hospice staff members (Table 1). Caregivers’ mean age was 61.1, ranging from 31 to 80; two-thirds were female (*n* = 20). Most of the caregivers had a bachelor’s degree or higher (76.7%) and were White (80.0%). Among the hospice staff were six nurses with varying roles (i.e., team manager, case manager, admission nurse, and long-term care facility education coordinator), two bereavement counselors, and one music therapist.

**Table 1:** Sample characteristics.

Characteristics	*M* (*SD*) or *n* (%)
***Caregivers (N = 30)***	
**Age, *M* (*SD*)**	61.1 (12.1)
**Sex, *n* (%)**	
Female	20 (66.7)
Male	10 (33.3)
**Ethnicity, *n* (%)**	
Hispanic or Latino	1 (3.3)
Non-Hispanic or Latino	29 (96.7)
**Race, *n* (%)**	
American Indian or Alaska Native	0 (0.0)
Black/African American	6 (20.0)
White	24 (80.0)
Asian American	0 (0.0)
Native Hawaiian or Other Pacific Islander	0 (0.0)
Other	0 (0.0)
**Education level, *n* (%)**	
Less than high school	0 (0.0)
High school or equivalent	2 (6.7)
Some college, associate degree	5 (16.7)
Bachelor’s degree	6 (20.0)
Graduate or professional degree	17 (56.7)
***Hospice staff (N = 9)***	
**Professional role, *n* (%)**	
Nurse	6 (66.7)
Bereavement counselor	2 (22.2)
Music therapist	1 (11.1)

*Note*. *M* = mean; *SD* = standard deviation.

Approximately half of the participants shared that they occasionally or frequently use AI tools, while the remaining half had never intentionally used AI. ChatGPT, Siri, and Alexa were commonly mentioned by participants who reported using AI, but most used these tools for personal purposes (e.g., writing, asking daily questions). Three family caregivers reported using AI tools for caregiving purposes: “*I just say, ‘Hey, Alexa, play ‘70s music for mom,’ and then they’ll put it right on. It’s good in that sense, or if I have a quick question, I can just say, ‘Hey, Alexa, look up anxiety issues for dementia patients,’ and they’ll regurgitate whatever it is that they come up with*” (Caregiver_15, Age 57). Another caregiver reported using ChatGPT to seek help interpreting their partner’s imaging results: “*This was as we were signing on to hospice. I did enter the entire MRI report into ChatGPT and asked for help interpreting it, and I’m not going to lie, it was right. Obviously, I talked to the doctors afterwards, but I was shocked by how detailed its interpretation was and the breakdown of how it defined words for me. It did it at a fairly readable level*” (Caregiver_25, Age 40). This participant also added that she used the list of questions generated by ChatGPT and took them to the doctor’s appointment. Two staff members reported using AI tools during their professional practice. One staff member told us ChatGPT had replaced Google as her main search engine and indicated that she uses it to ask about unfamiliar diagnoses: *“[I asked] ‘What things can I anticipate, as a hospice nurse, with somebody with this diagnosis?’ It gave me, ‘Well, I think you need to look at respiratory status.’ This may be an issue, that may be an issue. ‘Maybe these medications can be on board for that.’ […]—not perfectly, but enough for me to take from it and just be able to alter it. It has been very helpful in hospice, because, of course, I don’t know every disease process there is*” (Staff_01). Another staff member also shared her experience using AI features in a practice management software, which helped her write session summaries: “*Of course, you have to read over your notes after you’ve used it, and, yes, I do tweak them and everything, but it does save some time. It really does. It saves probably about 15 minutes per client*” (Staff_08).

To explore what impacts participants’ acceptance of AI in the context of hospice dementia care, we examined findings under the constructs of the TAM: attitudes toward AI, perceived usefulness, and perceived ease of use (Figure 1). In addition, our analysis identified an additional inductive category that impacted acceptance: the uniqueness of hospice. Several subcategories emerged within each category.

**Figure 1:**
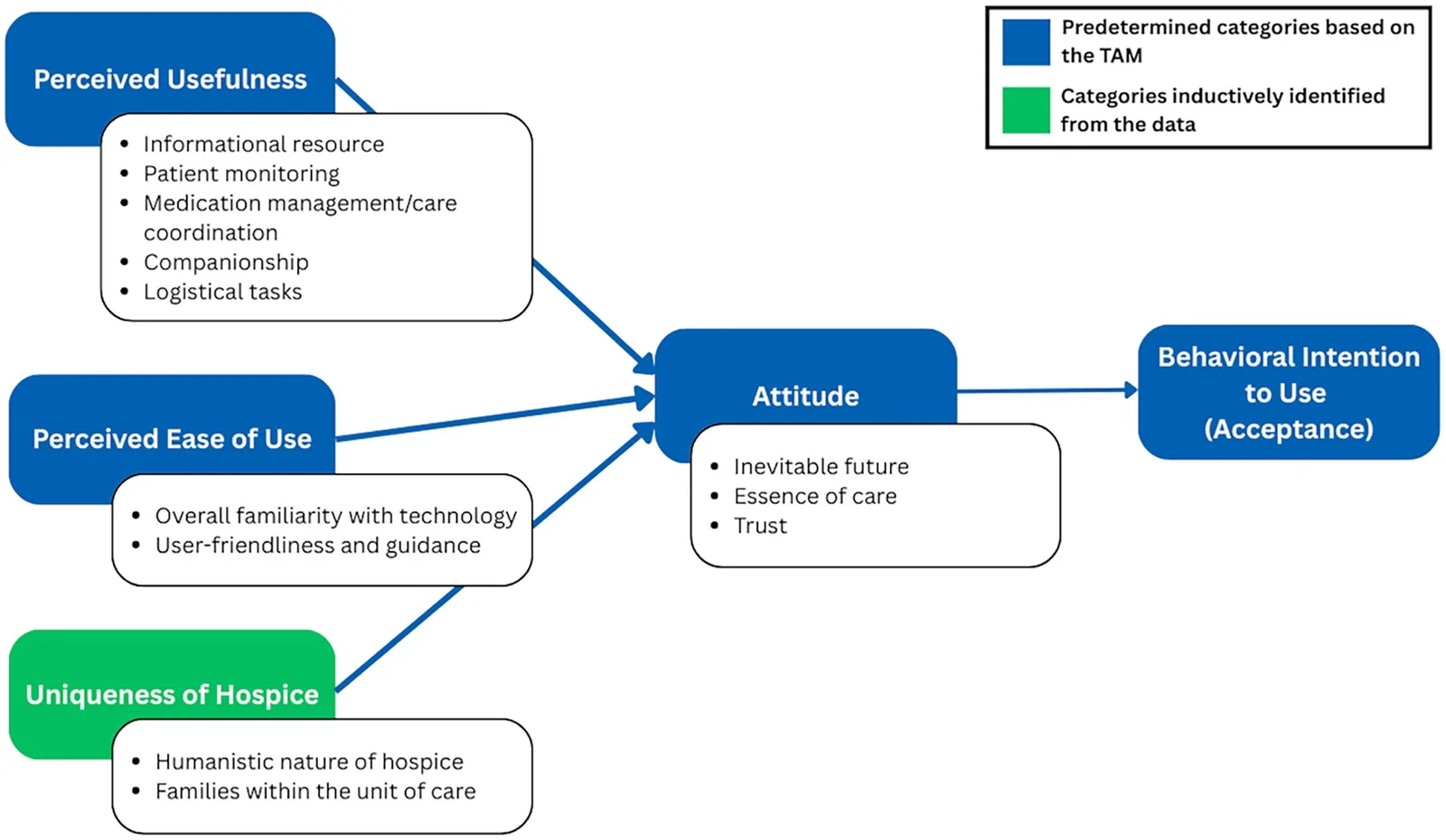
Categories and subcategories of study findings. *Note*. TAM = Technology Acceptance Model.

### Attitudes towards AI

Attitudes toward AI were defined as participants’ evaluative judgments regarding AI; three subcategories emerged.

#### Inevitable future

Participants perceived that AI will inevitably impact society and science, adding that we will need to be ready for its quick advancement. Some were positive toward this phenomenon (“*I’m sure that’ll be different, and it will be much more useful and ubiquitous*” [Caregiver_18, Age 71]), while others were not (“*I am scared what the world’s gonna look like in 5 years, 10 years, whatever, but who knows? We’ll see. Maybe they get a handle on it, and it doesn’t get spiraling and taking over everything”* [Caregiver_23, Age 65]). One caregiver saw AI as simply another resource, similar to the internet: “*I think I would view it as I would any other tool. At the inception of hospice would be good, or if it subsequently is developed during the time that my father, for example, is in hospice, then I would be receptive to then as well*” (Caregiver_03, Age 69). Another caregiver explained that she believed AI applications should be “*a starting point,*” not “*the end game*.”

#### Essence of care

As participants discussed AI integration in healthcare and hospice, several key components of care were highlighted as qualities for which human interaction should not be neglected. Empathy was discussed by some: “*Though it can try to mimic emotion, I don’t think it will come off as a genuine emotion. How can they?*” (Staff_01). A music therapist added that AI tools could observe external behaviors, but such approaches do not capture the emotional experience of being with someone. The need for “*reading the room*” was also discussed in terms of interpreting people’s facial expressions and body language; this was echoed by other staff and caregivers who explained that human health is so nuanced. Particularly with persons with dementia, a hospice nurse shared experiences of needing to interpret patients’ true intentions by consulting families about the patient’s history and wondered whether AI could provide such a holistic understanding beyond verbatim interpretation. A bereavement counselor also highlighted that, for persons with dementia, tailored strategies, such as redirecting, are needed instead of showing automatic empathy.

#### Trust

Participants commonly discussed concerns reflecting a lack of trust in AI tools, which were rooted in concerns about privacy. The ubiquity of AI tools, which enables tracking and listening to conversations, worried many, with some not using Siri or Alexa for this reason: “*I don’t, because I don’t trust them, because I think somebody’s collecting data on me*” (Caregiver_12, Age 71). However, another participant felt that AI does not add additional privacy risk because their medical records are already electronic: “*Everything is just open to a privacy breach at some point. I think AI just falls in the same category*” (Caregiver_22, Age 51).

Trust was also impacted by uncertain sources of information generated by AI and not knowing what is real: “*We probably see AI and we don’t even know it. I think that’s the dangerous part, because I’m like, ‘Well, what’s real then?’*” (Caregiver_12, Age 71). Concerns about hallucinations, inaccurate outputs, and the use of low-quality data were also discussed as factors that undermined participants’ trust in AI tools.

Trust in the humans behind AI technology was commonly brought up as well: “*Who runs those programs? Would it be Meta? Would it be—I don’t know—a reputable company? I don’t know. These are things I’m thinking about. Would it be some Joe Schmo sitting in his basement?*” (Caregiver_02, Age 53). Participants noted that AI tools could perpetuate human biases and expressed concerns about programmers lacking ethical integrity: “*Because people are so mean, especially today. And me take a response from an AI that was programmed by somebody that probably didn’t give that much about human beings? Oh, heck no. No!*” (Caregiver_08, Age 72). One caregiver expressed a lack of trust in humans exploiting the technology by prioritizing profit over genuinely helping people. Another caregiver mentioned she preferred the tool to be university-based and evidence-based to ensure its safety. Recommendations coming from the hospice team or peers were reported to increase caregivers’ trust in new AI tools: “*I think if the hospice team presented, this is a valuable resource for you, here’s how you use it, I would trust them because they almost gave their seal of approval*” (Caregiver_27, Age 59).

### Perceived usefulness

Perceived usefulness was defined as how helpful and beneficial participants believed AI tools could be in providing care for hospice patients with dementia; five subcategories emerged.

#### Informational resource

Receiving answers from AI in real-time and not having to read extensive materials were perceived as benefits, with one caregiver referring to AI as “*a great librarian to answer your question.*” Information needs included questions about pain management, dementia-related symptoms, medications, and what to expect throughout hospice. While conventional informational resources, such as pamphlets, were also discussed, caregivers reported wanting help in streamlining resources and clinical information tailored to their specific situation. One caregiver described noticing a change in their loved one’s breathing and not knowing what to do, and she shared her thoughts on how AI could have been helpful in that situation: “*I was videotaping his breathing to show the nurse. I was working on his breathing at night. I could, in theory, have not waited and taken the video [and] uploaded it to AI [and asked], ‘Should I be concerned about this because it doesn’t sound good?’ And maybe gotten an answer faster*” (Caregiver_25, Age 40). Beyond clinical information, one caregiver noted that she would feel more supported if AI tools could find stories or anecdotes of others in similar situations.

#### Patient monitoring

Monitoring physiological or behavioral changes of persons with dementia and identifying subtle changes were seen as valuable features. One caregiver noted, “*That would take a lot of the limits of my own knowledge, my own medical knowledge, my own expertise, my own practical knowledge, it would support that and improve my ability to take care of my family*” (Caregiver_11, Age 71). Another caregiver gave an example of his sister sleeping a lot. He had to go online to ask whether this was normal and agreed that tracking significant changes in her sleep would be helpful. AI was also perceived as helpful in generating alerts for families, overcoming the challenge of not always being physically near the patient. However, two caregivers stressed that they still wanted a human provider to assess and validate AI’s judgment of their loved one’s condition.

#### Medication management and care coordination

Caregivers perceived AI as having the ability to consolidate information about their loved one’s medication and appointments in one place and keep track of hospice staff visits and relevant documentation, describing it as a potential “*personal secretary.*” For instance, one caregiver shared her experience interacting with many healthcare personnel at home and thought having tools to help stay organized would have been helpful: “*Say, for instance, the nutritionist came, [and] I jotted down everything she said in my notes. It’s just in my notes in my phone. If I had some type of order, [like]—okay, the nutritionist is over here, the physical therapist is here, the occupational therapist is here, the nurse is here. If I could’ve had it, it would’ve been much easier if it wasn’t all jumbled together*” (Caregiver_09, Age 62). As some caregivers had to give medications to their loved one multiple times a day with varying doses, reminders were discussed as potentially helpful. However, three caregivers did not see a need for AI, as they indicated that they were able to keep track of scheduling and medications manually or with existing technology and thought it required human input anyway.

#### Companionship

Using generative AI tools to support companionship for persons with dementia and/or caregivers was a controversial topic among participants. Some staff commented that interactive tools that could respond to persons with dementia may be helpful, particularly if they could be programmed to be patient with them. One caregiver shared similar sentiments regarding robot pets: “*My dad, as he lost language, he became a little sillier. Some people didn’t know quite what to do with it, but I don’t think a robot pet would mind*” (Caregiver_16, Age 58). However, a hospice nurse also highlighted that extra caution is warranted for persons with dementia, as patients may easily confuse the generated outputs with reality. One caregiver expressed concern that persons with dementia might exhibit violent behaviors; while caregivers can calm them down, he was unsure how feasible it would be for AI-powered robots to do so without human warmth. Two staff members highlighted the potential value of integrating AI into entertainment tools for persons with dementia, such as games or simulations. However, a hospice nurse noted that if such tools were personalized to individuals’ cognitive or functional abilities, it would raise the question of how and by whom those levels of dementia severity would be determined.

Many caregivers also shared that they themselves experienced loneliness, frustration, and anxiety throughout hospice: “*Having a voice that they [families] could even interact with, either [to combat] loneliness or else because they have a concern. Yeah, I would say companionship certainly is something that AI could provide for many people*” (Caregiver_07, Age 77). Another caregiver described herself as a “*boiling pot all the time*” and commented that she would appreciate any outlet that allowed her to express herself. However, some caregivers and hospice staff were strongly opposed to AI offering companionship due to the lack of authentic human interaction: “*Find a real person because that relationship will be a lot more helpful and rewarding*” (Caregiver_19, Age 55).

#### Logistical tasks

Participants expressed interest in engaging AI to help with ordering supplies or doing repetitive daily chores. One caregiver noted: “*I would rather computers and AI do the mundane tasks. Clean the house. Take out the trash. Why do I have to do it?*” (Caregiver_28, Age 56). Similar sentiments were shared by hospice staff. One staff member commented: “*You easily know what needs to be reordered, but why […] does it have to be a licensed professional to do that ordering? Sometimes we just need a couple more hands to help us be more efficient to be able to be back in front of the patient and family more. I feel like the ordering of supplies, in particular, [could be done by AI]*” (Staff_03). Such perceptions were coupled with concerns about AI’s impact on job security. While staff welcomed AI assistance with operational tasks to increase productivity, they did not want AI to be trained to perform their roles in providing actual patient and family care.

Staff also discussed AI tools that could assist with charting. Reducing time and generating care strategies were mentioned as benefits, but staff emphasized that reviewing AI’s output was important to ensure autonomy and control. Organizing patients’ medical history and offering a quick summary were also seen as ways in which AI could be helpful. However, some expressed concerns about AI technologies listening to conversations with persons with dementia: “*I think we’ve all heard a lot of people with dementia say some pretty off-the-wall things. I would hate for some of that to go into notes without understanding what was happening in real life. I would hate for ChatGPT or any program to be like, ‘Oh my God. There’s abuse going on*’” (Staff_05).

### Perceived ease of use

Perceived ease of use was defined as participants’ perceptions of how effortless it would be to learn and use AI tools; two subcategories emerged.

#### Overall familiarity with technology

Participants felt that AI’s perceived ease of use was rooted in how familiar individuals were with technology in general. One caregiver commented: “*Someday, people in my position will be more experienced growing up with the internet from when they were small, and it’ll just seem second nature to do that. A book seems second nature to me. Artificial intelligence isn’t second nature*” (Caregiver_01, Age 80). While age was often mentioned, participants did not always assume that older generations were less tech-savvy. For instance, one participant, who did worry about older generations’ ability to use AI, added that education level also mattered: “*I also know I run across people who you think are younger and they’re like, ‘I don’t know how to do technology’*” (Caregiver_19, Age 55). Two participants mentioned their familiarity with social media applications when explaining their perceptions regarding AI’s ease of use: “*I wasn’t even doing Facebook until two weeks ago. [I was] practically tricked into it. I’ve never used Twitter. Those are pretty basic for most of the population of the United States. I’m not gonna be a pioneer in AI.”* (Caregiver_01, Age 80).

#### User-friendliness and guidance

Participants felt that the user-friendliness of AI tools was important: “*Maybe if I found it over my head, too technical, not user-friendly, or things that I just simply didn’t want to do. Yeah, I don’t want to do that.*” (Caregiver_10, Age 78). For individuals less familiar with digital technologies, one caregiver suggested it would be helpful if they could speak to the AI tools and ask questions without needing to type or use a specific search engine.

Caregivers also reported needing instructions and demonstrations prior to using AI tools: “*If there was a specific use for it that had been established and there was just a brief instruction. They say, ‘Okay, we use AI for this; this is how you do it.’ Yes, I could learn that quite easily*” (Caregiver_30, Age 51). One caregiver emphasized that AI tools for caregivers should not require significant time to learn to use, given caregivers’ already demanding schedules: “*Even though I’m basically home most of the week, there’s always something to do. I’m a one-woman show around here”* (Caregiver_07, Age 77). One caregiver also suggested that clear parameters should be established defining both the limits of AI and the distinct responsibilities of patients, families, and hospice providers: “*AI could do health education, it could do support. But there’s got to be a fine line of what a nurse does, or what a chaplain can do, what the roles are. […] there’s got to be parameters of what the limits are, because AI cannot—to the best of my knowledge, doesn’t have a moral pulse”* (Caregiver_12, Age 71). ‬‬‬‬‬‬‬‬‬‬‬‬‬‬‬‬‬‬‬‬‬‬‬‬‬‬‬‬‬‬‬‬‬‬‬‬‬‬‬‬‬‬‬‬‬‬‬‬

### Uniqueness of hospice

Participants brought up unique aspects of hospice that greatly impacted their thoughts toward AI being used for hospice patients with dementia; two subcategories emerged.

#### Humanistic nature of hospice

Both hospice staff and caregivers described human interaction, emotional support, empathy, and compassion as important characteristics of hospice. Those who associated AI with robots were particularly opposed to AI replacing human touch. For instance, one caregiver acknowledged the utility of machine learning for administrative tasks but rejected AI for companionship or answering questions, calling it “*false*”: “*In the space of hospice and the impact and human connection that you need to answer questions of where you are at a time, I do not believe that AI can provide any assistance to a family in that way. A robot is not a human being. Losing that human connection at a time when human connection is so critically important, because this decision-making is so impactful—I can’t imagine, and I won’t engage with a non-human in that regard. I’ll reject it all the time*” (Caregiver_04, Age 65). Another caregiver commented that, while AI tools may be appropriate for gaining factual information, they may not be subtle enough to handle emotional reactions in hospice: “*My immediate concern or thought with that is that conversations with caregivers or anyone who is in hospice have so much potential to veer toward an emotional reaction—even when people don’t think that that’s what’s gonna happen. They think they’re going to talk about I don’t know what—insurance—and it can very, very quickly—all of a sudden the grief and the whatever is brewing, can reach an emotional level*” (Caregiver_05, Age 50). Two caregivers were concerned that families who are already depressed or isolated might feel more disconnected and unheard by receiving responses from chatbots. A music therapist also discussed her thoughts on datafication: “*Everything already is data, I understand, in a way, but I also think, ‘Oh, hospice. Wait, don’t let it corrupt the sweet, super interpersonal nature of hospice care. I would hate to see it mechanized*” (Staff_09).

#### Families within the unit of care

Participants, particularly hospice staff, emphasized that the hospice philosophy views families, rather than patients alone, as the unit of care and that families face numerous challenges throughout the hospice experience that could be complicated by the use of AI tools. For instance, when discussing AI algorithms for identifying potential hospice candidates, two hospice nurses commented that hospice is a sensitive and emotional topic and were concerned that such applications might not be well received by patients’ families, potentially leading them to think providers had given up on them: “*I think families would view this as really dystopian, especially the families that are completely and totally in denial of everything. They would have a very hard time with this […] it might create confirmation bias amongst caregivers*” (Staff_08). Hospice staff commonly described family-related issues they encountered in their practice, such as the need to frequently repeat teaching with different family members or even spouses with cognitive impairment, supporting caregivers experiencing anticipatory grief, and working with caregivers in complex circumstances. Staff reported that these unique, family-related aspects of care should be considered when exploring the feasibility of integrating AI into hospice.

### Preparing for the future

Based on participants’ narratives, we identified actionable recommendations for designing and implementing AI tools in hospice dementia care; these were grouped into three subcategories: population-related, AI feature-related, and deployment-related (Table 2). Population-related recommendations addressed considerations regarding the unique characteristics of persons with dementia; AI-feature-related recommendations covered specific attributes participants thought were particularly important to be comfortable with AI tools; and deployment-related recommendations pertained to regulation, staff training, and timing within hospice.

**Table 2:** Recommendations for designing and implementing AI tools in hospice dementia care.

Recommendation	Relevant quotes
*Population-related*	
Do not consider persons with dementia as a monolithic population	“*There’s not like a cookie cutter type of dementia [….] We just have to be mindful that there’s just so many different aspects of dementia and so many different levels*” (Staff_01)
Provide strategies that align with the cognitive capacity of persons with dementia	*“With dementia, some of the things I have heard dementia people say are not things they ever would’ve said when their brain was healthy. I don’t want them to be encouraged through empathy to do something like, ‘I gotta catch a train.’ Boy, don’t we hear that a lot? ‘I gotta go catch a train. I gotta get the kids off the bus.’ I don’t want anyone being empathetic towards that. I want them to redirect”* (Staff_04)
Involve family caregivers into the design phase	“*I would always want to keep in mind the caregiver’s perspective as well as the patient, because the patient might benefit from AI. We also forget that the caregiver also usually has PTSD from [caring for the] patient with dementia*” (Staff_02)
*AI feature-related*	
Ensure the tool complies with the Health Insurance Portability and Accountability Act	“*My concerns would be the accuracy of it, the HIPAA [protection]. Can it be hacked? Can somebody come in and hack it and change things?*” (Staff_01)
Design the tool to be financially accessible to caregivers	“*If it was a high cost, that would be a huge deterrent for me, yeah. The cost would definitely play into it”* (Caregiver_22, Age 51)
Ensure that outputs generated by the tool are accurate and reliable	“*As long as they got their server farms working correctly and getting correct information, it’ll be good*” (Caregiver_23, Age 65)
Ensure the tool does not add too much time, effort, or responsibility for caregivers	“*I would be more concerned about ‘Is this going to take more of my time and more responsibility on my part?’*” (Caregiver_06, Age 78)
Ensure users understand how AI systems operate and the tool’s relevance to hospice populations	“*We would need to know where the information goes, who has access to it, how the algorithm was created, and did it look at our population?*” (Staff_08)
Enable human involvement throughout the user-AI interaction when necessary	“*It [AI] could be an asset and say, ‘What do you need help with?’ or ‘Are you frightened? Do you need somebody to talk to?’ and then have something built into that algorithm of like ‘We need to call the nurse*’” (Caregiver_12, Age 71)
*Deployment-related*	
Integrate staff’s perspectives throughout the design and implementation	“*[Engage] not just people who sit in offices and read books and magazine articles but actually people who are out with their feet on the ground, who see people every day […] to help develop the technology so that […] you see all sides of what our patients potentially could need and benefit from, if there is some benefit*” (Staff_06)
Generate evidence on how AI tools impact measurable hospice outcomes	“*Like, did that trial show that yes, it did improve time management and patient satisfaction? Then I would be okay integrating it. Otherwise I would be like, I don’t know about this*” (Staff_02)
Designate a team focused on AI ethics to provide oversight of deployment	“*Ethics should be at every single part, at every table, for any AI discussion at any time because it’s too—this stuff is too big, and it has access to too much, especially in healthcare*” (Staff_04)
Be transparent to patients and families about the scope, risks, and benefits	“*In order to feel comfortable using AI, I would want to make sure that it was explicitly known by the patients and families that that was what was happening. Ideally, I would like for it to be a choice so patients could say, ‘I don’t want that’ or ‘I’m okay with that’”* (Staff_09)
Offer basic AI training and education to staff	“*I also feel like maybe there’s space for some kind of education, especially for our generation, where it’s, ‘Here’s some basics’ so that you can feel like you have some kind of foundation or some trust in a certain technology if it’s gonna be used in practice*” (Staff_08)
Introduce AI-powered supportive tools to caregivers at the beginning of hospice	“*Well, I’d certainly wanna know that it’s available at first. There is an AI option that you can use to go check these things out: ‘Here’s the link if you wanna use it. This is available to you, and further down the road, we’ll give you more tools so that you can access it easier’*” (Caregiver_29, Age 64)

Abbreviation: AI = artificial intelligence.

## Discussion

Our findings show that caregivers and hospice staff are beginning to use AI-powered tools, most commonly Alexa and ChatGPT, to answer clinical questions, interpret medical data, and provide entertainment for persons with dementia. As the broader societal impact of AI-generated tools continues to grow,^26^^,^^27^ our findings confirm that such influence also extends to end-of-life caregiving contexts. However, our participants’ attitudes regarding trust and the ability of AI to provide meaningful, genuine care were nuanced, extending beyond considerations of beneficial features and ease of use.

Our participants viewed AI tools as being particularly helpful for assistive tasks, such as offering factual information and monitoring subtle changes in patients’ health, which aligns with emerging fields of research on AI-based technologies in dementia care.^28-^^30^ However, beyond these instrumental tasks, there were conversations about the emotional vulnerability of dementia caregivers during hospice and various thoughts on how AI technology might address social isolation and loneliness. While some caregivers and staff were resistant to the idea of using AI tools for companionship, emphasizing that such aspects of care fundamentally require human connections, others viewed these tools as potential sources of support. Although previous studies have explored the potential of AI applications to support social well-being,^31^^,^^32^ the tensions observed among our participants reflect a deeper philosophical question: which aspects of patient care can be delegated to AI technologies? AI tools may improve efficiency and productivity, and there have been studies demonstrating that responses generated by AI can be perceived as empathetic.^33^ Yet it remains unclear whether such human-AI interactions can be perceived as genuine or whether they might risk further disconnecting families during the profoundly human and vulnerable experience of caring for a dying loved one. In Rhee et al.’s study on the ethical implications of AI tools in palliative care, stakeholders similarly expressed that aspects such as clinician-patient trust, intuition, and nuance that enable holistic patient care could not be replaced by AI.^34^ Particularly in hospice dementia care, our participants emphasized that interactions with persons with dementia require extra attention, including efforts to seek meaning beyond literal interpretations, draw on a holistic understanding of the person, and focus on redirection. These requirements raised questions about whether AI tools could become sophisticated enough to provide this level of attention.

Participants also discussed streamlining appointment and medication schedules as AI features that could be potentially helpful. Such findings are consistent with research conducted by Wolfe et al., which reported that, when older adults were asked about caregiving-related AI features, 89% wanted appointment reminders and 75% were interested in daily medication reminders.^35^ Such tasks can indeed be accomplished using existing technologies without AI tools or even manually, and several studies have pointed out that older adults may be less likely to trust AI tools and automation.^36^^,^^37^ However, participants’ emphasis on these features suggests that additional support is needed beyond conventional methods to better incorporate new caregiving tasks into daily routines and commitments.

Staff also suggested using AI technology to automate administrative and logistics tasks, such as ordering supplies, allowing more time for direct patient care and family support. Such perceptions highlight that, despite ongoing social and ethical debates on AI integration in healthcare, there is a strong desire for tools that enable families and staff to focus on more meaningful interactions with care recipients. However, Hamer et al., who focused on the ethical implementation of AI-based prognostication tools in palliative care, found that AI automation may inadvertently lead to staff burden by identifying a large volume of consults, which, when intertwined with limited palliative care resources and clinicians, could actually lead to lower quality care.^38^ Together, these findings underscore that the value of AI tools lies not in automation itself but in whether implementation is designed to protect and enhance time for staff and caregivers to spend in direct and meaningful interaction with patients.

We also found that participants’ views on perceived ease of use were largely impacted by their previous experiences and familiarity with general technology. Even among those who were not familiar with AI tools, many caregivers were willing to learn to use them if they were supported by user-friendly design and proper guidance. Such findings are consistent with those of Nguyen et al., who reported that user-friendliness and repeated exposure to technology, alongside professional support, were critical for fostering comfort in using technology in palliative care.^39^ We also found that recommendations from hospice staff or peers amplified caregivers’ trust in AI tools, consistent with Chan et al.’s finding that older adults felt more confident adopting new technology when they were supported by tech-savvy peers and had access to professional assistance.^40^ Hence, our findings suggest that sustained adoption of AI tools among hospice caregivers requires infrastructure that provides accessible learning support through trusted social or professional channels.

Other noteworthy findings pertained to recommendations derived from our participants’ narratives. When asked about the optimal timing for introducing new AI tools in hospice, many caregivers suggested the beginning of hospice, even though this period is known to be emotionally challenging and overwhelming.^41^ Caregivers indicated that, if the burden required to use supportive tools were minimal, they would appreciate being introduced early on to resources that could enhance their caregiving experiences. Particularly given that some patients receive late hospice referrals,^42^ introducing tools later could therefore limit caregivers’ opportunities to benefit from them. Regarding AI features, beyond general considerations of cost, data accuracy, and transparency, which have been widely discussed in previous literature,^43^^,^^44^ our participants strongly emphasized the ethical integrity of human operators. Formal safeguards such as ensuring HIPAA (Health Insurance Portability and Accountability Act)-compliant tools and incorporating ethics committees were recommended, as well as ensuring the ethical conduct of humans developing and overseeing these technologies. Particularly for generative AI tools that allow users to actively enter data, it would be critical to establish clear boundaries between general clinical queries and the entry of identifiable patient information. Because today’s AI technology can generate human-like responses, images, and videos, participants’ considerations of trust extended beyond privacy and accuracy to include the potential for exploitation in blurring the boundaries between what is real and artificial. We also identified a strong desire among staff to be involved in decisions regarding AI integration into practice, driven in part by the anticipated potential challenges of being expected to use AI tools that carry professional responsibilities without sufficient understanding of technology. These sentiments align with Ulrich et al.’s argument that professionals should be trained not only to use AI tools but also to be involved in and evaluate the ethical appropriateness of their application.^45^

This study has several limitations. More than half of the caregivers we interviewed had graduate or professional degrees, so their perspectives may not be transferable to individuals with lower levels of education or digital literacy. Additionally, hospice staff were recruited from a single agency, which may limit the diversity of participants’ scopes of practice and patient populations, as well as contextual norms around technology use. Hence, future studies across multi-site settings are warranted to enhance transferability. Moreover, while our hospice staff encompassed professionals from various backgrounds in nursing, social work, and music therapy, other team roles such as hospice physicians, chaplains, or medical directors may have had different perspectives about AI. Finally, selection bias may have been introduced in our sample, as caregivers who participated in our study represent those who are willing to participate in research. Caregivers who experience substantial challenges in end-of-life caregiving may not have had the capacity to participate.

## Conclusion

We explored how families and professionals caring for persons with dementia in hospice view AI applications in the end-of-life context. Participants identified several AI features as helpful, including providing quick informational responses, offering outlets for interaction, and delivering personalized reminders. They also highlighted factors that make it easier to use AI tools, such as sustained exposure to technology, user-friendly interfaces, and clear guidance. Yet, caring for a dying loved one with dementia is an emotionally challenging and profoundly human experience. Participants emphasized that some aspects of care, such as empathy and trust, were complex and multi-layered; although AI tools may improve efficiency and productivity, participants emphasized that end-of-life care encompasses humanistic elements they preferred not to have replaced or delegated to technology. Given the rapid pace of AI advancements, participants’ quotes highlight the importance of ensuring that the perspectives of those at the frontline of care guide future directions. As one participant suggested, AI applications can serve as a “*starting point*” but should not be considered the “*end game.*” AI tools should be driven by the needs of their target population rather than innovation alone, particularly during vulnerable moments at the end of life.

## Supplementary Material

igag105_Supplementary_Data

## Data Availability

The parent study, which included the caregivers in this study, was registered with ClinicalTrials.gov (NCT05336344). The focus group interviews with hospice staff members were not preregistered. De-identified data may be made available upon request to the authorship team.
