# Supplementary material for: Is there a role for artificial intelligence in end-of-life care for persons with dementia? Perspectives of and recommendations by caregivers and hospice staff
Source: Innov Aging. 2026 Sep 18;10(11):igag105. doi: 10.1093/geroni/igag105 (PMC13695354; doi:10.1093/geroni/igag105)
Supplement: Supplementary file 1 [file igag105_supplementary_data.docx]

***Innovation in Aging* Supplementary Material: Oh, Oliver, Washington, & Demiris. Is there a role for artificial intelligence in end-of-life care for persons with dementia? Perspectives of and recommendations by caregivers and hospice staff.**

**Supplementary Table 1.** Interview guides

**Supplementary Table 2.** Reflexivity statement

**Supplementary Table 1.** Interview guides

Interview guide (Caregivers)

1. You may have heard the term “artificial intelligence” or “AI.” What comes to mind when you think about AI?

*Probe:*

- *For example, imagine if a computer or a robot could answer questions or interact with you just like a real human does. What do you think about that?*

2. Artificial intelligence is when computers or machines perform tasks that would generally require human brain power. Have you ever heard of or used ChatGPT or any other AI chatbots? Do you use Siri or Alexa? Have you had any experience with monitoring sensors or a social robot?
3. Now, imagine if there were a chance to use AI to help with your hospice experience. For example,

- AI could monitor and predict [patient name]’s changes in symptoms or behaviors throughout hospice.
- Chatbots could answer any questions you have or help you learn about hospice, caregiving, or [patient name]’s health at any time.
- AI could help with administrative tasks like scheduling or keeping track of [patient name]’s medications and symptoms.
- If you get overwhelmed or lonely as a caregiver, AI robots or chatbots could offer companionship by initiating conversations.

(If the interviewee previously said they have never used AI, skip the following question):

4. Can you share with me any **experiences of using AI** after starting hospice?

*Probes:*

- *What was the purpose?*
- *What did you think about it? (advantages, disadvantages, things that could be improved or new features that could be added)*

5. What are **your thoughts** on using AI to support patients and families in hospice?

*Probes:*

- *Do you think it would be useful for you as a caregiver in any way? Why or why not?*
- *Do you think it would be easy for you to learn and use AI?*
- *Can you describe any specific scenarios where you think AI could be helpful for you?*

6. If given the chance, would you be **willing** to learn more about AI and use it throughout hospice? Why or why not?

*Probes:*

- *What, if at all, would discourage you from using AI in hospice? (examples: cost, accuracy, continuity, access, equity, privacy, etc.)*

*(Additionally for people who are open to using AI)*

- *What information would you need to start using AI? (example: how this can help caregiving, how to use it, how to access it, how to ensure privacy protections, etc.)*
- *When would be a good time for you to learn? For example, would you prefer to learn about AI at the beginning of hospice, or would you need some time to adjust before receiving new information?*

Interview guide (Hospice staff)

I would like to give you a bit of a background information on AI just to make sure we are on the same page. To start with the definition, artificial intelligence or AI is when computers or machines perform tasks that would generally require human brain power. The most trendy and famous examples of AI now are ChatGPT or other generative AI chatbots. Siri and Alexa are also considered AI. Robots and monitoring sensors in our Apple watches also incorporate AI algorithms and features.

1. Now, I am going to provide you with a list of possible domains in which AI could be integrated in hospice care for patients with dementia. For each example, please rate how you feel about it from 0 to 5 (0 being the worst and 5 being the best). After rating these broad examples, we’ll discuss your thoughts overall.
a. **Monitor health information to predict outcomes**: For example, we could have algorithms identify potential candidates for hospice based on predictions of mortality or specific symptoms or behaviors.
b. **Generative AI embedded in the electronic health record**: Such a tool could draft your emails or texts to care recipients, or it could hear your conversations with patients/families and draft progress notes for you.
c. **Educational purposes**: AI could adjust the reading levels of long documentations or consent forms to make it easier for patients/families to understand the material, or patients/families could use chatbots or virtual coaches to ask questions or learn specific things about hospice, dementia, and caregiving.
d. **Conversational agent:** Chatbots could mimic the roles of a therapist or counselor and offer support to patients/families.
e. **Robots:** Robots with AI features could assist with daily tasks of caregiving for patients with dementia (e.g., handwashing, dressing, bathing), or social robots could act as pets to address loneliness.
2. From the list in Question 1, it seems like (fill in blank) was the domain you felt best about with regard to AI integration, and (fill in blank) was the domain you felt worst about. Let’s talk more about these. What prompted you to give these ratings?

*Probes:*

- What are some **benefits** you perceive related to these domains, if any?
- What are some **concerns** you have related to these domains, if any?

3. Beyond these broad examples, can you describe specific scenarios, if any, in which you might see AI being used in your care for patients with dementia?
4. Let’s imagine that [name of hospice agency] is going to integrate AI features into their system and have algorithms do certain tasks we just talked about. Under what conditions would you be **willing to be on board** with that decision?

*Probe:*

- What would you need to know more about?

5. What are some things that are important for you to make AI **useful** in your practice, especially when caring for patients with dementia?

*Probe:*

- *What information would you need to start using AI?*

6. What would be important for you feel **comfortable** and to find it **easy to use** AI in your practice?

*Probe:*

- *What, if at all, would discourage you from using AI?*

7. Suppose an AI engineer comes up to you and says, “*I’m brainstorming some AI tools I can develop to benefit hospice patients with dementia. What are some key things about this population that I should be aware of?*” How would you answer the engineer?

8. What was the most important thing you said or heard during this interview?

**Supplementary Table 2**. Reflexivity statement

As the leading author, I enter this work with a deep commitment to ensure that advanced technology and AI tools are driven by the needs and voices of patients and families that the healthcare system ultimately aims to serve. I pursue a future where AI is designed, developed, and deployed *with* PwD and families, rather than *to* or *for* these populations. I am a female PhD candidate in Nursing with a clinical background as a nurse and research experience in qualitative studies. I am fully aware that my previous experiences with AI and older adults may impact how I perceive and interpret the data.

To mitigate potential bias and enhance trustworthiness, I analyzed and discussed the data with the study’s senior and last author who is a PhD-prepared gerontologist and informatics researcher. The findings and written work were also shared and critiqued by my coauthors who bring expertise in palliative and hospice care and family caregiving, to ensure the findings were accurately grounded in the data.
